# Sweat and Sebum Preferences of the Human Skin Microbiota

**DOI:** 10.1128/spectrum.04180-22

**Published:** 2023-01-05

**Authors:** Mary Hannah Swaney, Amanda Nelsen, Shelby Sandstrom, Lindsay R. Kalan

**Affiliations:** a Department of Medical Microbiology and Immunology, School of Medicine and Public Health, University of Wisconsin, Madison, Wisconsin, USA; b Microbiology Doctoral Training Program, University of Wisconsin, Madison, Wisconsin, USA; c Department of Medicine, Division of Infectious Disease, School of Medicine and Public Health, University of Wisconsin, Madison, Wisconsin, USA; d M. G. DeGroote Institute for Infectious Disease Research, David Braley Centre for Antibiotic Discovery, Department of Biochemistry and Biomedical Sciences, McMaster University, Hamilton, Ontario, Canada; Lerner Research Institute

**Keywords:** human microbiome, sebum, skin, skin microbiome, sweat

## Abstract

The microorganisms inhabiting human skin must overcome numerous challenges that typically impede microbial growth, including low pH, osmotic pressure, and low nutrient availability. Yet the skin microbiota thrive on the skin and have adapted to these stressful conditions. The limited nutrients available for microbial use in this unique niche include those from host-derived sweat, sebum, and corneocytes. Here, we have developed physiologically relevant, synthetic skin-like growth media composed of compounds present in sweat and sebum. We find that skin-associated bacterial species exhibit unique growth profiles at different concentrations of artificial sweat and sebum. Most strains evaluated demonstrate a preference for high sweat concentrations, while the sebum preference is highly variable, suggesting that the capacity for sebum utilization may be a driver of the skin microbial community structure. In particular, the prominent skin commensal Staphylococcus epidermidis exhibits the strongest preference for sweat while growing equally well across sebum concentrations. Conversely, the growth of Corynebacterium kefirresidentii, another dominant skin microbiome member, is dependent on increasing concentrations of both sweat and sebum but only when sebum is available, suggesting a lipid requirement of this species. Furthermore, we observe that strains with similar growth profiles in the artificial media cluster by phylum, suggesting that phylogeny is a key factor in sweat and sebum use. Importantly, these findings provide an experimental rationale for why different skin microenvironments harbor distinct microbiome communities. In all, our study further emphasizes the importance of studying microorganisms in an ecologically relevant context, which is critical for our understanding of their physiology, ecology, and function on the skin.

**IMPORTANCE** The human skin microbiome is adapted to survive and thrive in the harsh environment of the skin, which is low in nutrient availability. To study skin microorganisms in a system that mimics the natural skin environment, we developed and tested a physiologically relevant, synthetic skin-like growth medium that is composed of compounds found in the human skin secretions sweat and sebum. We find that most skin-associated bacterial species tested prefer high concentrations of artificial sweat but that artificial sebum concentration preference varies from species to species, suggesting that sebum utilization may be an important contributor to skin microbiome composition. This study demonstrates the utility of a skin-like growth medium, which can be applied to diverse microbiological systems, and underscores the importance of studying microorganisms in an ecologically relevant context.

## INTRODUCTION

Human skin is a unique environment for microbial colonization. The skin is desiccate, nutrient poor, and slightly acidic, which together contribute to a hostile microbial niche that is unwelcoming to pathogen colonization. However, the skin harbors a unique community of microorganisms, termed the skin microbiome, that thrives on the skin and has adapted to these seemingly harsh skin conditions (1). Rather than simply occupying the skin niche, the skin microbiota carry out a plethora of beneficial functions, contributing to numerous aspects of skin health and function that range from the enhancement of the skin’s physical barrier to the early-life education of the immune system (2–6). As such, a mechanistic understanding of how the microbiome and its members carry out these critical functions is necessary for a full understanding of the role that the microbiome plays in our health and for the development of therapeutics to mitigate skin disorders associated with a disrupted microbiome. For these reasons, the development of model systems to study skin microorganisms in the laboratory under ecologically relevant settings is needed.

In the laboratory, skin isolates are traditionally grown and studied in rich medium that supplies the microorganism with a wealth of nutrients to support rapid and abundant growth. However, nutrient-rich medium is not representative of the nutrient availability found on the skin, which is limited to minimal host-derived nutrients and metabolites from sweat, sebum, and corneocytes (1). Previous studies have demonstrated that skin commensals utilize nutrients available on the skin, for example, through the lipase-mediated degradation of sebum lipids by Cutibacterium acnes to promote bacterial adherence (7–9), the enzymatic cleavage of sphingomyelin to promote barrier integrity and microbial colonization (2), and the presumed incorporation of lipids into the bacterial cell envelope (10, 11). Furthermore, recent efforts to study skin-associated pathogens in synthetic skin-like media versus traditional rich media have revealed that the observed phenotypes are highly dependent on the medium type (12, 13). Therefore, the use of culture conditions that mimic those of the human skin is critical for translating findings between the laboratory setting and the native skin environment.

In addition to overcoming low nutrient availability on the skin, the skin microbiota must also adapt to variations in this nutrient availability across different skin sites, which is a result of the physiologically distinct skin microenvironments (sebaceous, moist, dry, and foot). These skin microenvironments exist as the result of variations in sweat and sebum gland densities and distributions as well as differences in occlusion (exposure to the environment) (14–16). For example, sebaceous sites (i.e., the face and back) are characterized by higher densities of hair follicles and sebaceous glands (14), while moist sites (i.e., the belly button and the bend of the elbow) can have more abundant and active sweat glands, which lead to increased moisture levels (15, 17). Numerous studies have demonstrated that the microbial communities in these distinct microenvironments are unique in their composition and function (18–21), suggesting that the observed community differences between microenvironments are in part due to the differential microbial utilization of available nutrients and/or microbial inhibition by human skin secretions.

To more accurately mimic the native skin environment in the laboratory, we developed a custom artificial human skin secretion medium for cultivating skin commensals that includes physiologically relevant compounds found in both sweat and sebum. Because the microbial communities across the biogeography of the skin are driven by distinct microenvironments, we hypothesize that skin-associated bacteria isolated from these microenvironments will exhibit differential growth in sweat and sebum. Using 15 phylogenetically diverse bacterial skin strains isolated from healthy skin and the skin pathogen Staphylococcus aureus, we show that each strain has a unique artificial sweat and sebum preference profile. We find that overall, skin-associated bacterial species have a strong preference for high sweat concentrations. In contrast, the preference for sebum varies, with strains exhibiting a range of lipophilic to lipophobic phenotypes. Overall, our findings provide additional evidence to support the hypothesis that the skin microbial community structure varies across skin microenvironments as a result of differences in skin secretions and nutrient availability. Understanding microbial nutrient preferences under physiologically relevant conditions is a critical step toward developing model skin microbiome systems and interrogating microbial interactions.

## RESULTS

To systematically study skin commensal growth preferences in nutrients representative of those found on the skin, we developed artificial sweat and sebum that can be supplemented to minimal medium in a dose-dependent manner. We developed this minimal medium (termed basal medium here) to provide the minimal nutrients required to support the growth of most skin-associated bacteria. The composition of the basal medium is based on M9 minimal medium consisting of salts, glucose as a carbon source, and additional select amino acids and vitamins (see Table S1 in the supplemental material). For example, we found that l-proline, nicotinic acid, and pantothenic acid, described previously as additions to S. aureus minimal medium (22), were necessary for Staphylococcus growth. Similarly, biotin has been identified as a vitamin required for certain *Corynebacterium* species (23, 24), and cobalt is added to allow vitamin B_12_ biosynthesis by select skin-associated corynebacteria (25).

### Artificial sweat development.

Human eccrine sweat is a complex mixture of solutes that is secreted from eccrine sweat glands, which are the most numerous sweat glands found across the body’s surface area and are responsible for the highest volume of sweat production (26). Eccrine sweat, composed of mainly salt water, contains a variety of other solutes in various concentrations, including lactate, urea, glucose, bicarbonate, amino acids, electrolytes (in addition to Na^+^ and Cl^−^), and vitamins (26, 27). To develop artificial sweat that is consistent with the composition of human eccrine sweat and is suitable for microbial growth, we developed a formulation considering previously described synthetic sweat media (12, 28) and extrapolating eccrine sweat constituents and their concentrations reported in the literature (Data Set S1). The composition of the artificial sweat used in this study is listed in Table 1.

**TABLE 1:** Artificial sweat composition^*a*^

Artificial sweat component	Composition
Salts	
Sodium chloride	2 g
Sodium pyruvate	50 mg
Monosodium phosphate, anhydrous	10 mg
Calcium sulfate dihydrate	50 mg
Potassium H carbonate	170 mg
Carbon source	
d-Glucose	20 mg
Amino acids	
Glycine	50 mg
l-Leucine	25 mg
l-Serine	80 mg
l-Alanine	40 mg
l-Arginine	50 mg
l-Histidine	100 mg
l-Valine	20 mg
l-Isoleucine	15 mg
l-Lysine HCl	35 mg
l-Phenylalanine	15 mg
l-Tyrosine	20 mg
l-Glutamine	5 mg
l-Aspartic acid	25 mg
l-Glutamic acid	35 mg
l-Proline	8 mg
l-Ornithine HCl	50 mg
l-Citrulline	50 mg
l-Tryptophan	20 mg
l-Asparagine	10 mg
l-Threonine	35 mg
Others	
Creatinine	5 mg
Lactic acid (88%)	0.5 g
Urea	0.5 g

a Values are expressed in milligrams or grams (per liter of medium).

### Artificial sebum development.

Human sebum is a complex mixture of lipids that is secreted from sebaceous glands, which are distributed across the body and most densely located on the scalp and face (29). Sebum is composed of triglycerides, free fatty acids, squalene, wax esters, cholesterol esters, and cholesterol (30). To provide a sebum-like lipid source when culturing skin commensals, we developed artificial sebum similar in composition to that of sebum L, which was developed previously by Lu et al. and was found to be physiochemically consistent with human sebum (31). The composition of the artificial sebum used in this study is listed in Table 2. Because this lipid formulation is not miscible with water, we found that a ratio of 1 part artificial sebum to 3 parts Tween 80 allowed the solubilization of the artificial sebum in aqueous solution. Tween 80 is a nonionic surfactant that contains oleic acid (an abundant fatty acid present on the skin [32]) and is commonly used for the culture of skin microorganisms (33).

**TABLE 2:** Artificial sebum composition

Artificial sebum component	Composition (% [wt/wt])
Squalene	15
Cholesterol	1.2
Cholesterol oleate	2.4
Waxes	
Paraffin wax	10
Hexadecyl palmitate	15
Fatty acids	
Oleic acid	1.4
Myristic acid	2.5
Lauric acid	2.5
Palmitic acid	5
Triglycerides	
Olive oil	10
Coconut oil	10
Cottonseed oil	25

### Selection of bacterial isolates from diverse taxa and numerous skin sites.

**FIG 1:**
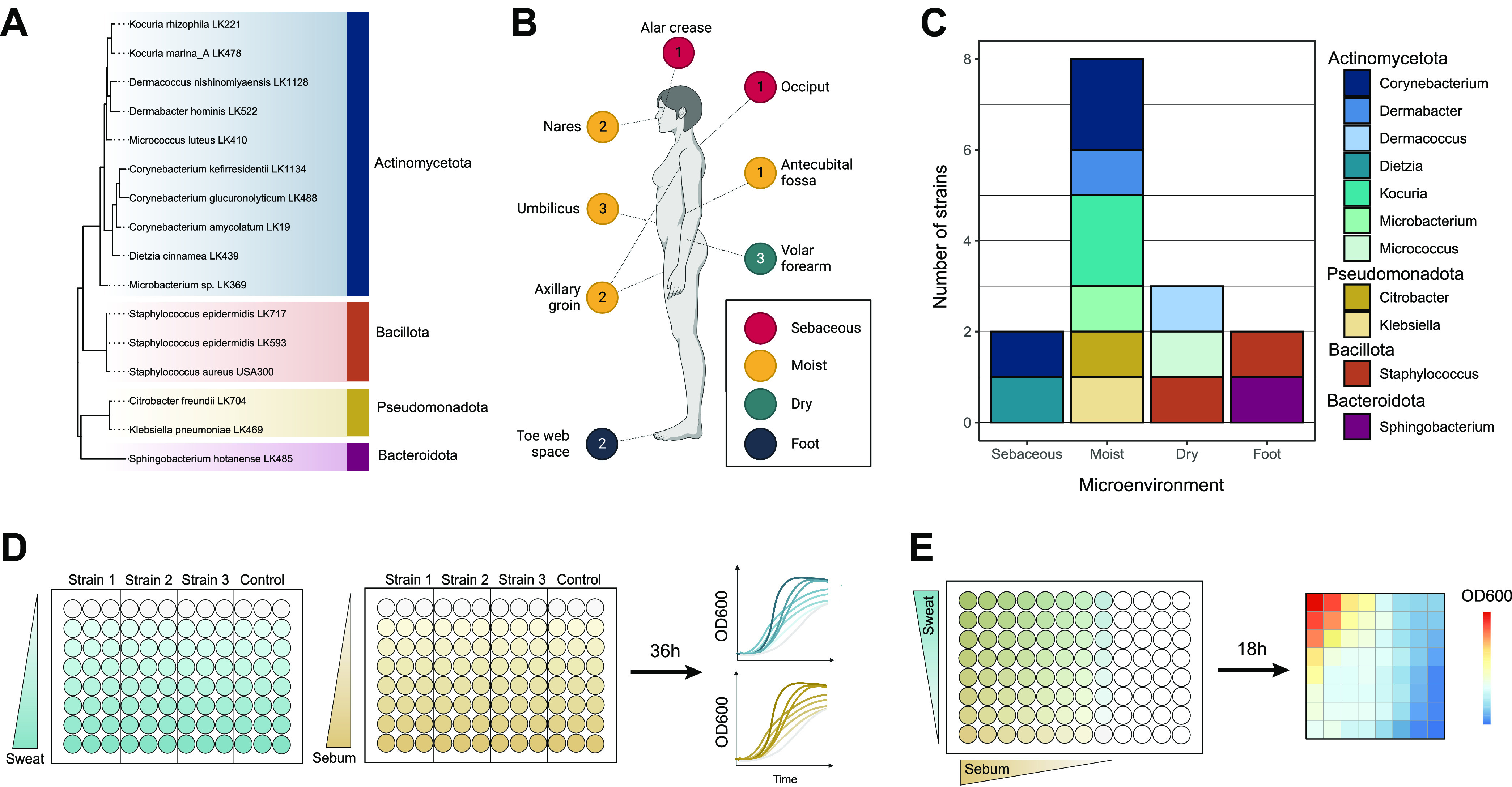
Overview of skin strains and experiment design. (A) Fifteen strains isolated from healthy subjects and Staphylococcus aureus USA300 were selected for use in this study and include representatives from the phyla *Actinomycetota* (blue), *Bacillota* (orange), *Pseudomonadota* (yellow), and *Bacteroidota* (dark pink). 16S rRNA sequences from each strain were aligned, and an approximately maximum likelihood phylogenetic tree was inferred. The tree was rooted using the archaeon Halobacterium salinarum. (B) Skin strains were isolated from 8 skin sites, and the number of strains isolated from each site is indicated. The axillary/groin site is a combined swab of the axilla and groin skin. Colored circles represent skin microenvironment categories: sebaceous (red), moist (yellow), dry (green), and foot (blue). (C) Numbers of strains isolated from each microenvironment and their respective genus taxonomic assignments. (D) Experimental overview of the growth curve assays. Per 96-well plate, three strains were inoculated into increasing concentrations of artificial sweat or sebum media and incubated at 37°C for 36 h. OD_600_ measurements were taken at even intervals. (E) Experimental overview of the checkerboard-like assay. Per 96-well plate, one strain (representing a single biological replicate) was inoculated into increasing concentrations of combined artificial sweat and sebum media. The plate was incubated at 37°C for 18 h, at which point an endpoint OD_600_ measurement was taken.

To evaluate the growth of skin commensals in media containing artificial sweat and sebum, we selected 15 phylogenetically diverse bacterial skin strains isolated from healthy skin in our laboratory, as well as the skin pathogen S. aureus, for testing. Strains were selected taking into consideration phylogeny, the skin microenvironment, and the body site from which they were isolated, with the goals of maximizing the taxonomic diversity of isolates from distinct body sites and including representatives from both high- and low-abundance members of the healthy skin microbiome. The selected skin isolates represent species from the four most abundant phyla present on the skin: *Actinomycetota* (*n* = 10 strains), *Bacteroidota* (*n* = 1 strain), *Pseudomonadota* (*n* = 2 strains), and *Bacillota* (*n* = 3 strains) (Fig. 1A; Data Set S2).

Furthermore, the isolate collection used in this study contains strains isolated from 8 distinct skin sites (alar crease, occiput, nares, umbilicus, antecubital fossa, axillary/groin, volar forearm, and toe web space), including representatives from the 4 following skin microenvironments: sebaceous (*n* = 2 strains), moist (*n* = 8 strains), dry (*n* = 3 strains), and foot (*n* = 2 strains) (Fig. 1B and C). We have observed that less abundant or rare species are more frequently isolated from moist skin sites; therefore, we selected numerous strains isolated from this microenvironment to increase the phylogenetic diversity of the isolates tested. All strains grow aerobically at 37°C, with most reaching the end of exponential growth within 36 h in rich media (Fig. S1).

For the following reasons, multiple strains were selected from the same genus or species for use in the study. Two Staphylococcus epidermidis isolates were selected to represent the high level of strain diversity exhibited within the species (34); S. epidermidis LK593, isolated from the volar forearm, falls within clade A and sequence type 73 (ST73), while S. epidermidis LK717, isolated from the toe web space, falls within clade A and ST7. Similarly, multiple species of the *Corynebacterium* genus were selected to represent the high level of genomic diversity found within the genus (25, 35), and two species of the *Kocuria* genus were selected to provide insight into this understudied low-abundance skin microbiome member (36, 37).

### Skin strains exhibit a preference for high sweat concentrations.

The 16 selected strains were then cultured for 36 h in basal medium containing increasing concentrations of sweat for growth curve analysis (Fig. 1D). Sweat concentrations were tested from 0× (only basal medium) to 4× (only artificial sweat and no basal medium). We selected concentrations above 1× artificial sweat in order to test the tolerance of the skin strains at higher salt and solute concentrations and to account for the variation in sweat compositions across individuals. These higher sweat concentrations may be physiologically relevant, as skin surface sweat rapidly evaporates and may leave residual sweat constituents, which has been observed previously during sweat collection (38–40).

**FIG 2:**
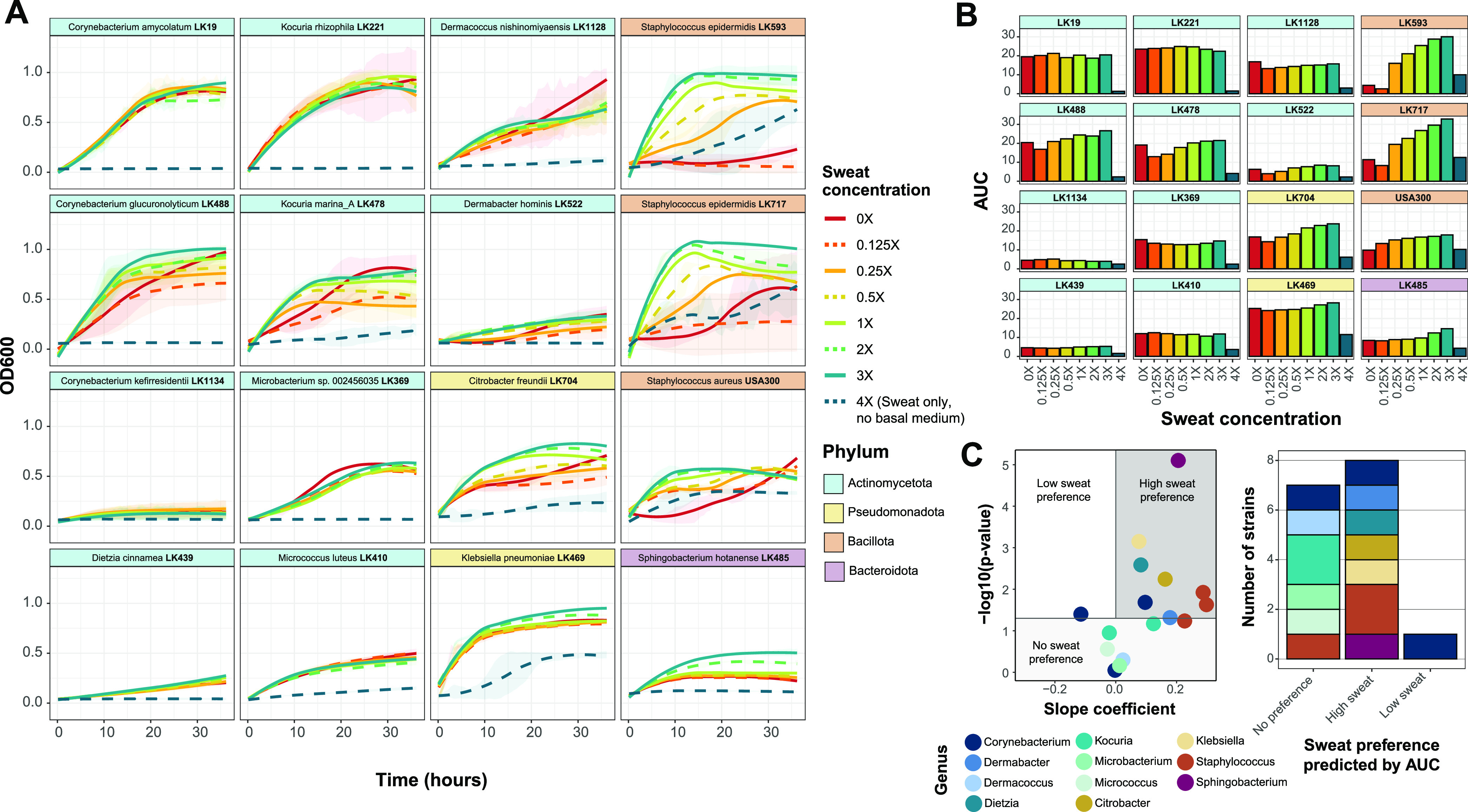
Skin species exhibit concentration-dependent growth in artificial sweat. (A) For each strain, growth curves were collected at eight concentrations of artificial sweat medium over 36 h. Strains are ordered by phylum-level taxonomic assignment. The 0× sweat medium contains only basal medium, and 4× sweat contains only artificial sweat. Curves were generated using LOESS for averaged OD_600_ values from at least 2 biological replicates with 3 technical replicates each. Ribbons represent the standard deviations across biological replicates. (B) The area under the curve (AUC) was calculated for each strain and each artificial sweat concentration. The strain order is identical to that in panel A. (C) Linear regression was performed with minimum-maximum-normalized AUC data. For each strain, the slope coefficient (β) and *P* values for the model are plotted. Strains are classified into sweat preference based on the following coefficient and *P* value cutoffs: low-sweat preference at a β value of <0 and a *P* value of <0.05, high-sweat preference at a β value of >0 and a *P* value of <0.05, and no sweat preference at a *P* value of >0.05.

We observed that generally, the skin strains tested exhibited either similar growth patterns at all sweat concentrations tested, apart from 4× sweat, or increased growth with increasing sweat concentrations (Fig. 2A). This concentration-dependent increase in growth is particularly evident for the two Staphylococcus epidermidis strains, which grew very poorly and variably in basal medium alone or with low concentrations of sweat. Furthermore, we observed minimal or no growth over time when strains were grown in 4× sweat, which contained no basal medium, suggesting that either certain basal medium components are required for rapid growth or the concentration of sweat solutes was too high or not well tolerated by the strains tested. To quantify the growth curves for each strain and sweat concentration tested, we calculated the maximum growth rate and the area under the curve (AUC), which integrates information about lag phase, growth rate, and carrying capacity (41). We observed either little AUC change across sweat concentrations (excluding 4× sweat) or a concentration-dependent increase for these two metrics (Fig. 2B; Fig. S2A). We confirmed this finding by performing a linear regression with the AUC data to assess the sweat preference of each strain, which revealed that most strains exhibit either no preference for sweat or a high-concentration preference (Fig. 2C).

We also tested the pathogen S. aureus in sweat medium, which revealed a sweat-concentration-dependent increase in growth, similarly to the S. epidermidis strains. However, unlike these commensal staphylococci, growth was comparatively much less robust at higher sweat concentrations, which supports the notion that skin commensals are highly adapted to the skin environment and available nutrients. Indeed, staphylococci have numerous osmoprotectant transport systems for providing resistance to changing osmolarity (42), which is likely exhibited on the skin as a result of the production and evaporation of sweat. It has been suggested that S. epidermidis may have an increased presence or expression of these protective systems compared to S. aureus (43).

### Skin strains exhibit a range of sebum concentration preferences.

**FIG 3:**
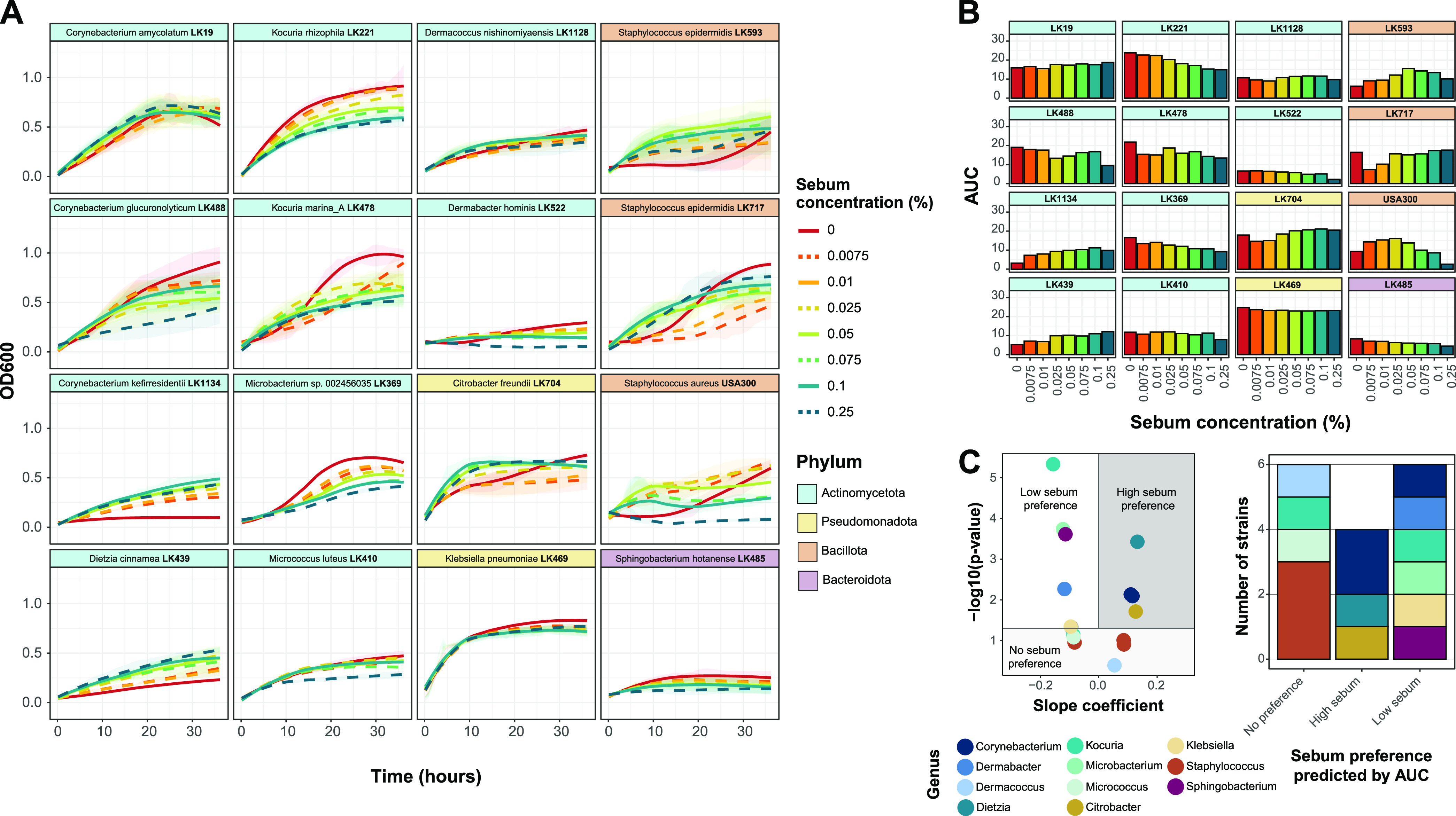
Growth in artificial sebum varies by skin species. (A) For each strain, growth curves were recorded at eight concentrations of artificial sebum medium over 36 h. Strains are ordered by phylum-level taxonomic assignment. Zero percent sebum contains only basal medium. Curves were generated using LOESS for averaged OD_600_ values from at least 2 biological replicates with 3 technical replicates each. Ribbons represent the standard deviations across biological replicates. (B) The area under the curve (AUC) was calculated for each strain and each artificial sebum concentration. The strain order is identical to that in panel A. (C) Linear regression was performed with minimum-maximum-normalized AUC data. For each strain, the slope coefficient (β) and *P* values for the model are plotted. Strains are classified into sebum preferences based on the following coefficient and *P* value cutoffs: low-sebum preference at a β value of <0 and a *P* value of <0.05, high-sebum preference at a β value of >0 and a *P* value of <0.05, and no sebum preference at a *P* value of >0.

To evaluate the growth of the skin strains in artificial sebum medium, sebum concentrations were tested from 0% (basal medium only) to 0.25%. We selected 0.25% as the maximum concentration because above this, the lipids reach their solubility limit. We found that the growth curve patterns with differing sebum concentrations varied drastically across strains. For example, certain strains, including Corynebacterium kefirresidentii and Dietzia cinnamea, exhibit a concentration-dependent increase in growth with increasing sebum concentrations, whereas other strains, such as Kocuria rhizophila and *Microbacterium* sp. strain LK369, exhibit a concentration-dependent decrease in growth (Fig. 3A). Furthermore, some strains, including Micrococcus luteus and Dermacoccus nishinomiyaensis, exhibited relatively similar growth curves regardless of the sebum concentration. These patterns are similarly reflected in the AUC and maximum growth rate for each strain and sebum concentration (Fig. 3B; Fig. S1B). We then classified the isolates by their sebum preference, determined by linear regression of the AUC data, into the following categories: low-sebum, high-sebum, or no sebum preference. We found that the skin strains demonstrate a range of sebum preferences, with multiple strains falling into each category (Fig. 3C).

### Growth profiles of skin strains in sweat and sebum cluster by phylum.

**FIG 4:**
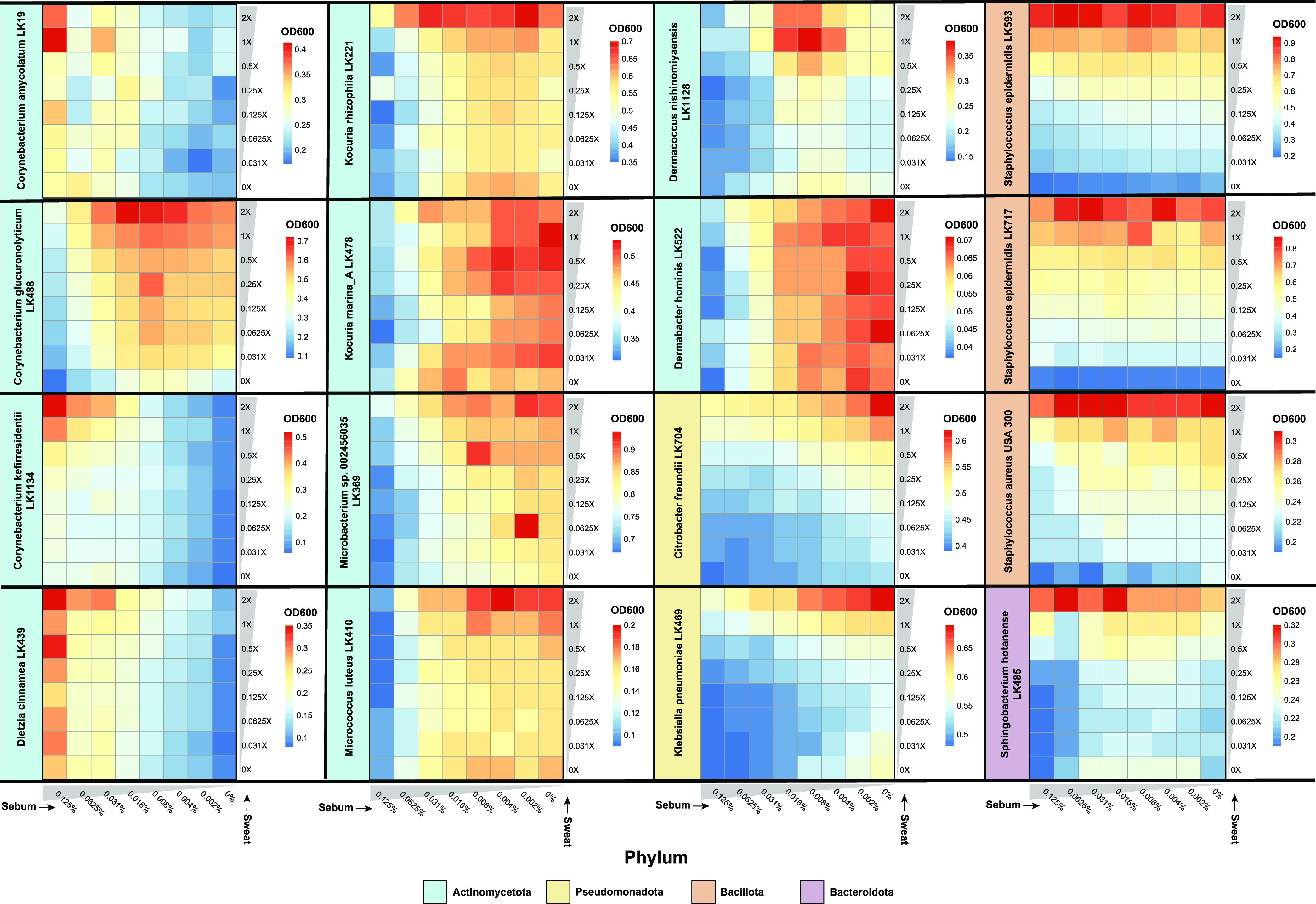
Sweat and sebum checkerboard assays reveal species-specific growth patterns. Strains were cultured in unique combinations of artificial sweat media (0×, 0.031×, 0.0625×, 0.125×, 0.25×, 0.5×, 1×, and 2×) and artificial sebum media (0, 0.002, 0.004, 0.008, 0.016, 0.031, 0.0625, and 0.125%) for 18 h, after which an endpoint OD_600_ reading was recorded. Data from at least three replicates were collected for each strain, and the OD_600_ values from uninoculated medium were subtracted from the experimental OD_600_ values. Average OD_600_ values for the replicates are shown.

To assess the growth of the skin strains in medium containing both artificial sweat and artificial sebum, we developed a checkerboard-like assay for testing strain growth at 8 concentrations of sebum (0% through 0.125%) across 8 concentrations of sweat (0× through 2×) (Fig. 1E). Strains were cultured for 18 h, after which an endpoint optical density at 600 nm (OD_600_) measurement was collected. We selected this time point because the growth curve data suggest that 18 h is when strains had reached early stationary phase and exhibited the largest differences in OD_600_ values among the various sweat or sebum concentrations (Fig. 2A and Fig. 3A). The checkerboard assay results demonstrate a wide range of growth phenotypes across the different sweat and sebum concentrations for each strain (Fig. 4; Fig. S3). In certain cases, highly similar growth patterns occur for phylogenetically related species (e.g., Citrobacter freundii and Klebsiella pneumoniae from the *Pseudomonadota* phylum or the three strains from the Staphylococcus genus). However, across strains from the *Actinomycetota* phylum, a range of growth patterns and sweat/sebum preferences exist. Even within a single genus, as for *Corynebacterium* and *Kocuria*, for which multiple species representatives are included, multiple phenotypes are demonstrated. We also found that strains with certain sweat or sebum preferences in the growth curve experiments (Fig. 2C and Fig. 3C) shifted their preferences when the secretions were combined, demonstrating the multifactorial impact of sweat and sebum on the growth of certain strains such as C. kefirresidentii and C. freundii.

**FIG 5:**
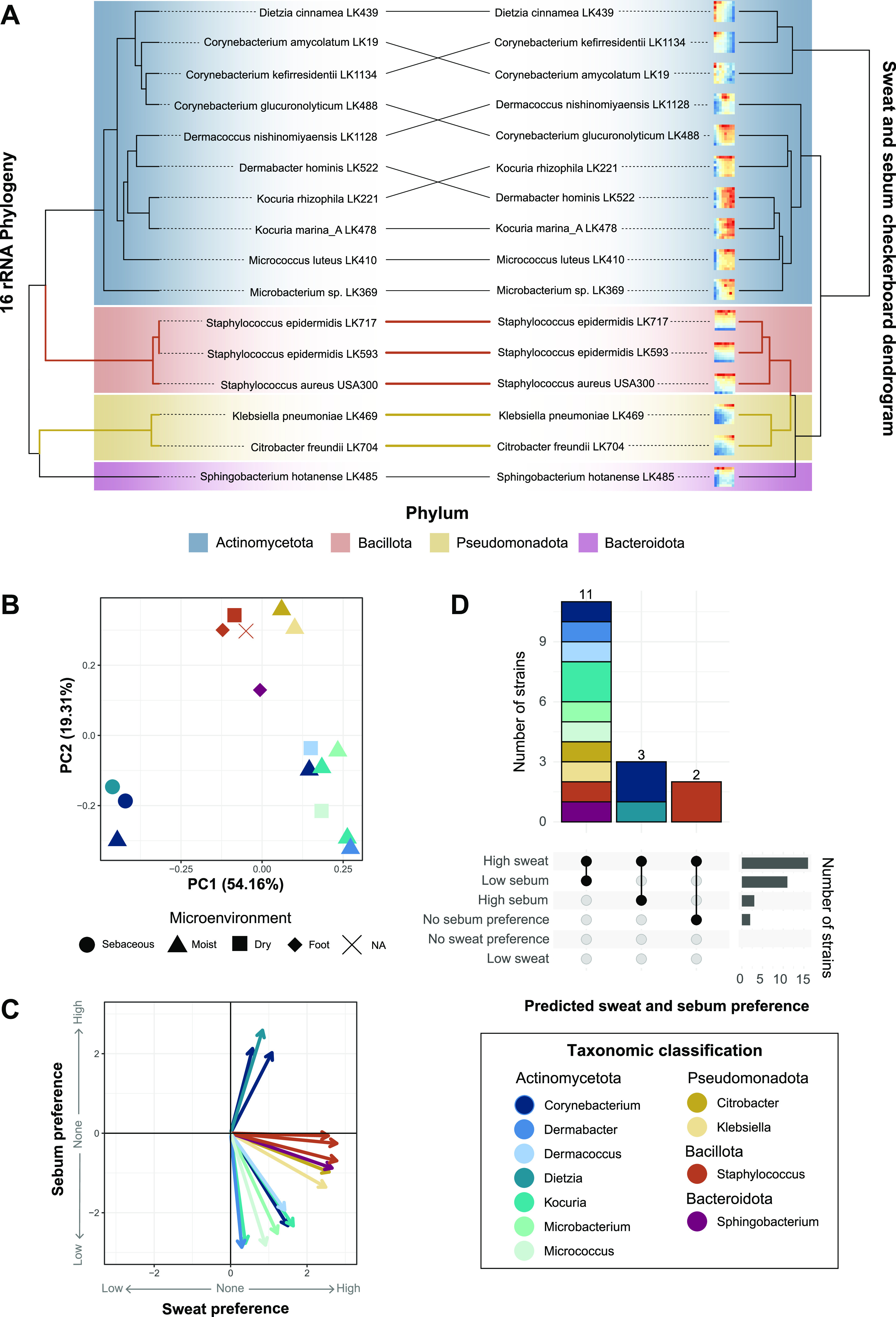
Skin species groups by sweat and sebum preferences. (A) Hierarchical clustering was performed on z-score-normalized data from the sweat and sebum checkerboard assays. Results are plotted as a tanglegram with the resulting dendrogram (right) and the 16S rRNA phylogeny from Fig. 1A (left). (B and C) Principal-component analysis (B) and multiple linear regression analysis (C) were performed using the z-score-normalized data. In panel C, the slope coefficients for sweat concentrations and sebum concentrations from the regression analysis are plotted as *x* and *y* coordinates, respectively. NA, not applicable. (D) Strains are classified by sweat and sebum preferences based on the following slope coefficient (β_Sweat_ or β_Sebum_) and *P* value cutoffs: low-sweat preference at a β_Sweat_ value of <0 and a *P* value of <0.05, high-sweat preference at a β_Sweat_ value of >0 and a *P* value of <0.05, no sweat preference at a *P* value of >0, low-sebum preference at a β_Sebum_ value of <0 and a *P* value of <0.05, high-sebum preference at a β_Sebum_ value of >0 and a *P* value of <0.05, and no sebum preference at a *P* value of >0. Strains are colored by phylum (A) or genus (B to D).

To identify groups of strains with similar nutrient preferences, we employed hierarchical clustering of the checkerboard data, which grouped strains with overlapping profiles into three main clusters (Fig. 5A). We further observed clustering by phylum, and this phylogenetic signal was supported by a tanglegram analysis, which demonstrated a correspondence between the checkerboard dendrogram and the 16S rRNA phylogeny for the *Bacillota* and *Pseudomonadota* subtrees. Within the *Actinomycetota* phylum, two clusters formed, with one cluster consisting of strains that exhibited increased growth with increasing sebum concentrations (e.g., Corynebacterium amycolatum) and the other cluster consisting of strains that exhibited decreased growth with increasing sebum concentrations (e.g., Kocuria marina). Consistent with this analysis, we performed principal-component analysis (PCA) and identified three distinct groupings, with two groups consisting of strains from the *Actinomycetota* phylum and the other consisting of strains from the *Bacillota*, *Pseudomonadota*, and *Bacteroidota* phyla (Fig. 5B). When considering the skin microenvironment, a strong grouping of strains by the microenvironment from which they were isolated was not observed, with the exception of the sebaceous-site-derived isolates of *C. kefirresidentii* and Dietzia cinnamea.

### Skin strains prefer high sweat concentrations but varied sebum concentrations.

To quantify the strength of the sweat and sebum preferences of each strain, we performed a multiple linear regression (MLR) analysis of the checkerboard data and ordination of the resulting slope coefficient (β) values, which represent the rate of change of the OD_600_ with increasing sweat or sebum concentrations. This revealed that most strains have a preference for higher sweat concentrations, with S. epidermidis LK717, S. epidermidis LK593, S. aureus, Sphingobacterium hotanense, and C. freundii demonstrating the strongest preferences for high sweat concentrations (Fig. 5C). For these strains, the sweat concentration had relative importance values of 99.94%, 99.15%, 94.15%, 90.17%, and 87.48%, respectively, in the regression model (Data Set S3). However, certain strains, including Dermabacter hominis and K. marina, appear to have relatively weaker preferences for sweat, with relative importance values of 1.02% and 1.91%, respectively. On the other hand, the sebum preferences varied drastically for each strain, with only C. amycolatum, D. cinnamea, and *C. kefirresidentii* exhibiting a preference for high sebum concentrations (relative importance values of 93.24%, 90.58%, and 77.78%, respectively). Both S. epidermidis strains exhibited little preference for any sebum concentration (relative importance values of 0.06% for LK717 and 0.85% for LK593). The remaining strains demonstrated a preference for low sebum concentrations, with D. hominis, K. marina, and M. luteus showing the strongest preferences for low sebum concentrations and relative importance values of 98.98%, 98.09%, and 90.47%, respectively, in the regression model.

Although we did not observe grouping based on the skin microenvironment, we were interested if the skin microenvironment from which the strains were isolated was reflected in the strengths of the sweat and sebum preferences. We observed that S. epidermidis LK717 and S. hotanense, both of which were isolated from the foot microenvironment (increased moisture and sweat levels), exhibited strong preferences for high sweat concentrations (Fig. S4). Furthermore, the two skin strains isolated from sebaceous skin sites, *C. kefirresidentii* and *D. cinnamea*, were 2 of the 3 strains overall that showed a preference for high sebum concentrations. We did not observe a pattern for the other isolates from dry or moist microenvironments.

The strains were then classified by their sweat and sebum preferences according to the MLR slope coefficients and corresponding *P* values. This revealed that 11 of the 16 strains exhibit high-sweat and low-sebum preferences, 3 strains exhibit high-sweat and high-sebum preferences, and 2 strains exhibit a high-sweat preference but no sebum preference (Fig. 5D). Overall, a range of artificial sweat and sebum preferences are exhibited by the skin strains, with a preference for high sweat concentrations being the most common.

## DISCUSSION

The study of microorganisms under conditions similar to those of their native environment is crucial for characterizing microbial physiology, ecology, and function. Particularly, the skin microbiota have adapted to the nutrient-limited conditions on the skin and, as such, are equipped to metabolize the available nutrients derived from sweat, sebum, and corneocytes present in the skin environment. Yet skin microorganisms are often supplemented with a surplus of nutrients when they are cultured in nutrient-rich media in the laboratory. In this study, we developed defined artificial sweat and sebum media that support the growth of diverse skin bacteria from the four most abundant phyla present on the skin. These artificial formulations contain physiologically relevant compounds that are found in human sweat and sebum and are based on numerous studies that have characterized the composition of human sweat and sebum (16, 27, 31, 44–53) as well as previously developed artificial formulations for the growth of microorganisms (12, 28).

Using the developed artificial sweat and sebum, we systematically tested the growth of 15 bacterial strains isolated from healthy human skin as well as the skin pathogen S. aureus. We found that the strains overall have a strong preference for media with high artificial sweat concentrations and generally exhibit more growth when supplemented with sweat. Eccrine sweat glands, which produce a salty secretion that our sweat formulation mimics, are distributed across almost all of the human body (26). Therefore, it could be expected that the skin microbiota have adapted to utilize this ubiquitous source of nutrients for their metabolism and growth. While, to our knowledge, no previous studies have systematically evaluated individual skin commensal growth dynamics under sweat-like conditions, Lam and colleagues cultured Staphylococcus, *Cutibacterium*, and *Micrococcus* skin isolates in filter-sterilized sweat collected from human subjects, revealing that Staphylococcus species metabolized sweat to produce malodor-associated compounds (54). Furthermore, Callewaert et al. were able to successfully use artificial sweat to culture skin microbial communities for up to 21 days, which closely resembled the native microbial community from which they were sampled (28). Thus, our study builds on previous knowledge to provide evidence that the skin microbiota utilize sweat as a nutrient source for metabolic function and growth.

In particular, we observed that the growth of S. epidermidis, and, to a lesser extent, S. aureus, was highly responsive to increasing sweat concentrations, suggesting that some constituent of the artificial sweat could be required or preferred for abundant growth. However, the exact component(s) of the artificial sweat that results in this concentration-dependent growth increase is unknown. It has been predicted that Staphylococcus and *Corynebacterium*, and likely other skin microbiota, utilize urea and amino acids in sweat as nitrogen sources and glucose, lactic acid, and amino acids as carbon sources (16, 55). Human sweat itself is considered to be hypotonic (~0.2% NaCl), but the salt content of the skin surface is likely much higher as a result of residual salt deposits after sweat evaporation. While the highest NaCl concentration tested in this study reached only 1.05% (4× artificial sweat) and is not considered a high salt concentration, many skin microorganisms are highly halotolerant and capable of withstanding drastic changes in osmotic pressure, including Staphylococcus and *Corynebacterium* species (55–61). In addition, nearly all of the other genera in this study have been identified as being halotolerant in previous reports (62–67).

In contrast to the observed preference for higher concentrations of artificial sweat in the skin strains tested, we found that the preference of the strains for artificial sebum concentrations varies, even among strains within the same genus. For example, certain strains, such as those from the *Corynebacterium* and *Dietzia* genera, exhibited lipophilic tendencies and showed a strong preference or even a requirement for higher sebum concentrations. This is likely a result of lipid auxotrophy, which is exhibited by certain microorganisms colonizing the skin, including several *Corynebacterium* species and fungal species within the *Malassezia* genus (1). For example, the human skin commensal Corynebacterium jeikeium lacks a fatty acid synthase and is thus dependent on exogenous fatty acids. Fatty acids are the building blocks for corynomycolic acids, the characteristic major constituents of the cell envelope of most *Corynebacterium* species (11), and are thus necessary for corynebacterial growth. Indeed, we observed that *C. kefirresidentii*, one of the most abundant species colonizing the skin (68), requires sebum for growth, suggesting that *C. kefirresidentii* is a lipid auxotroph. This is supported by a recent investigation of the *Corynebacterium* genus, which found that species lacking fatty acid synthase I, which is the case for *C. kefirresidentii*, tend to be lipophilic (35).

While some of the tested skin strains preferred sebum, many strains demonstrated inhibited growth with increased sebum concentrations. Sebum itself, and particularly the free fatty acids that make up a portion of this lipid-rich secretion, has antimicrobial properties, which could explain why many skin strains in this study have a low tolerance for sebum (32, 69, 70). Interestingly, over 80% of S. epidermidis strains can esterify fatty acids to cholesterol, which may protect them from sebum’s antimicrobial effects (55, 71). In our checkerboard assay, regardless of the sebum concentration, both S. epidermidis strains demonstrated similar growth patterns, and the sebum concentration had very little relative importance in the linear model for describing growth with increasing sweat and sebum concentrations. Therefore, certain skin commensals, including Staphylococcus species, may be well equipped to tolerate the antimicrobial properties of sebum on the skin.

An important function of the skin is protection against invading pathogens, which is contributed in part by the skin’s harsh physical landscape (1). We found that the skin pathogen Staphylococcus aureus exhibited relatively poor growth at high concentrations of sweat and sebum compared to its commensal relative Staphylococcus epidermidis, which thrived at high concentrations of these artificial skin secretions. Yet in rich media, S. aureus and S. epidermidis grow robustly and have very similar growth patterns. This further reinforces that skin commensals are highly adapted to utilize available skin nutrients and also provides evidence that sweat and sebum contribute to resistance to pathogen colonization on the skin.

The different microenvironments across the skin harbor distinct skin microbial communities (18). Presumably, these unique communities exist in part because of the physiological differences in the skin, including sweat gland, sebaceous gland, and hair follicle densities and distributions, which lead to differences in sweat and sebum secretion. Here, we demonstrate that skin isolates exhibit unique growth profiles in sweat and sebum and have specific sweat and sebum concentration preferences, providing *in vitro* evidence to support the hypothesis that the microbial community organization differs across skin microenvironments in part as a result of differences in skin secretions and nutrient availability. Our results further suggest that sebum may be a dominant driver of these differences, as sebum preferences differed drastically from strain to strain. Indeed, Pyle et al. found that mice deficient in stearoyl-coenzyme A desaturase 1, which causes the decreased synthesis of several major sebum components, showed significant microbial community differences compared to wild-type mice (72). Furthermore, sebaceous skin sites are typically dominated by microbial species that can thrive in this lipid-rich, antimicrobial environment. For example, the anaerobic, aerotolerant species Cutibacterium acnes metabolizes and breaks down sebum components to support its growth (73, 74) and to control the growth of other microbes (75), contributing to its success at colonizing sebaceous skin sites.

While careful consideration was taken during the development of the artificial sweat and sebum media to best represent these human skin secretions, some limitations exist surrounding this artificial system. For example, physiological factors such as sweat rate, which can impact the composition of sweat (40, 76), were not considered during medium development or experimentation. Furthermore, we did not incorporate keratinocyte-derived lipids when developing the artificial sebum formulation; while these lipids are a small fraction of the total extractable surface lipids (77), they are nevertheless likely to be accessible to and metabolized by the skin microbiota, particularly at skin sites with a low sebaceous gland density. Also, antimicrobial peptides produced by the host are present in skin secretions and undoubtedly influence microbial growth (78–80); therefore, our findings do not reflect the effect that these important molecules may also have on commensal growth and microbiome structure. It is also important to note that the addition of Tween 80 to aqueous preparations of the artificial sebum medium, while necessary for solubilizing the sebum, could influence skin commensal growth. Another limitation of this study is that only skin bacterial strains that grow aerobically were selected for testing. Oxygen concentrations vary on and in the skin, and certain prevalent and abundant skin commensals, including C. acnes, thrive under anoxic conditions (1). Therefore, future studies to interrogate how anaerobes utilize sweat and sebum are necessary. Finally, we did not assess the chemical stability of our artificial sweat and sebum formulations. However, we have observed consistent results using media stored at 4°C and protected from light over a period of approximately 2 months.

In conclusion, we have developed artificial sweat and sebum media that support the growth of a wide range of skin microorganisms. We also show that skin strains demonstrate unique growth profiles at different concentrations of sweat and sebum and have distinct preferences for these skin secretions. These findings provide evidence to support why different skin microenvironments, characterized by their physiological properties, harbor unique microbial communities. Importantly, our findings support the use of physiologically relevant media for the study of skin microorganisms. Future studies include further probing the impact of strain diversity on skin microbiota nutrient utilization, as well as using metabolomics to characterize the metabolic output of skin commensal growth in the artificial media and transcriptomics to examine pathways that may be involved in sweat and sebum utilization. These efforts will provide important insight into how the skin microbiota have adapted to thrive on the skin and how we can harness these adaptations in the context of health and disease.

## MATERIALS AND METHODS

### Bacterial strains and media.

The bacterial strains used in this study are part of the Kalan laboratory skin strain biobank and were isolated from the skin of healthy volunteers under an institutional review board (IRB)-approved protocol at the University of Wisconsin-Madison (Sandstrom S, Nguyen UT, Salamzade R, Swaney MH, Ludwikoski I, Wan H, Rybolt M, Safdar N, and Kalan L. Antimicrobial potential of the human skin microbiome. In progress). Strain information is listed in Data Set S2 in the supplemental material. For routine culturing, strains were struck out from frozen glycerol stocks onto brain heart infusion agar plates supplemented with 0.2% Tween 80 (BHITw80) and incubated at 37°C until single colonies of ~0.5 to 1 mm in size formed. Between 1 and 5 colonies were inoculated into 4 mL BHITw80 broth and incubated at 37°C with shaking (200 rpm) for 24 h for use in subsequent experiments.

### Basal medium composition and preparation.

A minimal medium was developed for use as a basal medium for the experiments in this study. A summary of the basal medium composition is listed in Table S1. As described previously, certain skin strains exhibit improved growth when a small amount of glucose is autoclaved in the presence of other medium components (20, 81). As such, 11.28 g/L M9 salts (Sigma-Aldrich) and 0.1 g/L glucose were prepared in an aqueous solution and autoclaved at 121°C with a sterilization time of 15 min. When cooled, the following medium components were added at the final concentrations indicated: 0.1 mM CaCl_2_, 2 mM MgSO_4_, 50 nM CoCl_2_, 100 mg/L l-arginine, 200 mg/L l-proline, 2 mg/L thiamine-HCl, 2 mg/L nicotinic acid, 2 mg/L calcium pantothenate, and 2 mg/L biotin. Additional glucose was added to bring the final concentration to 2 g/L. See “Artificial sweat development” and “Artificial sebum development,” below, for details regarding the addition of artificial sweat and sebum to basal medium.

### Artificial sweat development.

Artificial sweat was developed based on the composition of previously described synthetic sweat media (12, 28) as well as by the extrapolation of sweat constituents and their concentrations reported in the literature (16, 27, 44–53). As a range of concentrations exist for many constituents reported in human sweat, we selected values representative of the average concentration of each constituent. The final artificial sweat composition used in this study can be found in Table 1. To prepare the artificial sweat, all components indicated in Table 1 were added to H_2_O to the desired concentration, followed by filter sterilization and storage at 4°C. We have had success in preparing concentrated solutions of the artificial sweat up to an 8× concentration. Filter-sterilized artificial sweat was then added to the prepared basal medium after autoclaving (see “Basal medium composition and preparation,” above) to create a culture medium with the desired sweat concentration.

### Artificial sebum development.

Artificial sebum was developed based on the previously described artificial sebum L (31). Minor modifications were made based on the availability of individual components (the replacement of spermaceti with hexadecyl palmitate, the addition of myristic acid and lauric acid, and the exclusion of palmitoleic acid). The final composition used in this study can be found in Table 2. To prepare the artificial sebum, all components indicated in Table 2 were added to a glass bottle placed in a hot water bath. The components were heated with intermittent stirring until the solids melted to form a homogeneous oil. The final product was stored at 4°C. The method for the preparation of the sebum for use in culture medium is as follows. Artificial sebum and Tween 80 were heated to an oil consistency, followed by the mixing of 1 part artificial sebum with 3 parts Tween 80; this step is necessary for the solubilization of the artificial sebum in the culture medium. The sebum-Tween 80 suspension was then added along with H_2_O, M9 salts, and glucose during basal medium preparation, before autoclaving (see “Basal medium composition and preparation,” above). We have had success in preparing aqueous solutions of the artificial sebum at concentrations of up to 0.25%.

### 16S rRNA phylogenetic tree.

rRNA sequences were identified from the whole-genome sequences of the 16 strains using barrnap (v0.9). 16S rRNA gene sequences were extracted, aligned using muscle (v3.8.1551), and trimmed using trimal (v1.4.rev22). Fasttree (v2.1.10) was used to infer an approximately maximum likelihood phylogenetic tree of the 16S rRNA sequences. The tree was rooted using the archaeon Halobacterium salinarum.

### Sweat and sebum growth curve assays.

Eight concentrations of sweat medium (0×, 0.125×, 0.25×, 0.5×, 1×, 2×, 3×, and 4×) were prepared in basal medium using a concentrated stock of 4× artificial sweat. Similarly, eight concentrations of sebum medium (0, 0.0075, 0.01, 0.025, 0.05, 0.075, 0.1, and 0.25%) were prepared in basal medium using the artificial sebum-Tween 80 formulation described above. The 0× sweat and 0% sebum media are comprised of only basal medium, while 4× sweat is comprised of only concentrated 4× artificial sweat. The concentrations of basal medium components in the final medium preparations remained constant and were adjusted during medium preparation. A liquid culture of each test strain (see “Bacterial strains and media,” above) was diluted to an OD_600_ of 3.0, and 10 μL of the diluted culture was then inoculated into sweat or sebum medium in a lidded 96-well plate (CytoOne) to a final OD_600_ of 0.15 in a volume of 200 μL. We selected this OD_600_ because we have observed that many skin strains exhibit density-dependent growth, where too low of an inoculum results in minimal or no growth. The assay setup is outlined in Fig. 1D. The plate was incubated at 37°C with shaking in an Epoch 2 microplate reader (BioTek, USA) for 36 h, and OD_600_ measurements were recorded every 10 min. Data from three technical replicates and at least two biological replicates were collected for each strain and medium condition.

For each medium condition, the OD_600_ technical replicates for each strain were averaged and subtracted from the average of the OD_600_ technical replicates for the uninoculated medium. Averages and standard deviations were then calculated for all biological replicates for each strain and medium condition. A locally estimated scatterplot smoothing (LOESS) model was fit for the biological replicates, and the area under the curve (AUC) was calculated using the trapz function of the pracma R package (v2.3.8). The maximum growth rate for each strain and medium concentration was calculated with the all_easylinear function of the growthrates R package (v0.8.2). Linear regression analysis was performed with minimum-maximum-normalized AUC data, and the slope coefficient (β) and *P* values were used to determine sweat or sebum preferences. A β value of <0 and a *P* value of <0.05 indicate a low-sweat or a low-sebum preference phenotype, a β value of >0 and a *P* value of <0.05 indicate a high-sweat or a high-sebum preference phenotype, and a *P* value of >0.05 indicates no sweat or sebum preference.

### Sweat and sebum checkerboard assays.

4× sweat medium was prepared in basal medium from a concentrated stock of 8× artificial sweat. To prepare diluted sweat media for the checkerboard assay, 4× sweat medium was added to the top row (wells 1 to 8) of a 96-well plate, and basal medium was added to all remaining rows (wells 1 to 8) (Fig. 1E). Twofold dilutions were performed down rows A to G, with the last row consisting of only basal medium. Similarly, to prepare diluted sebum media for the checkerboard assay, 0.25% sebum medium was added to the first column of another 96-well plate, and basal medium was added to the remaining columns (wells 1 to 8) (Fig. 1E). Twofold dilutions were performed across columns 1 to 8, with the last column consisting of only basal medium. Ninety-five microliters from each well of both the sweat and sebum medium dilution plates was transferred to another 96-well plate, resulting in a sweat-and-sebum checkerboard containing 190 μL of medium per well.

A liquid culture of each test strain (see “Bacterial strains and media,” above) was diluted to an OD_600_ of 3.0, and 10 μL of the diluted culture was then inoculated into all wells of the sweat-and-sebum checkerboard plate. Plates were incubated at 37°C with shaking (200 rpm) in a New Brunswick Innova 42 incubator (Eppendorf) for 18 h. After incubation, cells were mixed by pipetting, and endpoint OD_600_ measurements were taken in an Epoch 2 microplate reader (BioTek, USA). Data from at least three replicates were collected for each strain.

The OD_600_ values for each plate were subtracted from the average of the OD_600_ replicates for uninoculated medium. Averages and standard deviations were then calculated for all replicates of each strain. Data were z-score normalized for subsequent analyses. For each strain, a multiple linear regression analysis was performed using the formula OD_600_ ~ sebum concentration + sweat concentration, and the relative importance values of sweat and sebum concentrations in the model were determined using the calc.relimp function of the relaimpo R package (v2.2-6). Principal-component analysis and hierarchical clustering (average linkage method) were performed, followed by tanglegram analysis of the resulting dendrogram and 16S rRNA phylogeny using the tanglegram function of the dendextend R package (v1.15.2). For multiple linear regression analysis, the slope coefficients (β_Sweat_ and β_Sebum_) and *P* values were used to determine sweat and sebum preferences. A β_Sweat_ or β_Sebum_ value of <0 and a *P* value of <0.05 indicate a low-sweat or a low-sebum preference phenotype, a β_Sweat_ or β_Sebum_ value of >0 and a *P* value of <0.05 indicate a high-sweat or a high-sebum preference phenotype, and a *P* value of >0.05 indicates no sweat or sebum preference.

### Quantification and statistical analysis.

The R statistical package was used to generate figures and perform statistical analyses.

### Code availability.

The code for performing the analysis and generating the figures is available on GitHub (https://github.com/Kalan-Lab/Swaney_etal_SkinMediaPaper).

### Data availability.

Genome assemblies are publicly available from the NCBI under BioProject accession number PRJNA803478. Genome assembly accession numbers for all strains are provided in Data Set S2 in the supplemental material.
