# Supplementary material for: Sweat and Sebum Preferences of the Human Skin Microbiota
Source: Microbiol Spectr. 2023 Jan 5;11(1):e04180-22. doi: 10.1128/spectrum.04180-22 (PMC9927561; doi:10.1128/spectrum.04180-22)
Supplement: Supplemental file 1 — Supplemental material. Download spectrum.04180-22-s0001.pdf, PDF file, 1.2 MB [file spectrum.04180-22-s0001.pdf]

Supplemental Table S1. Basal medium composition.

Basal Medium Component	Composition (mg)	Final Concentration
Salts		
M9 Salts (g)	11.28[a] (g)	11.28 g/L
Cobalt Chloride hexahydrate (ug)	12[b] (ug)	50 nM
Calcium Chloride anhydrous	11[b]	0.1 mM
Magnesium Sulfate heptahydrate	490[b]	2 mM
Carbon Sources		
Glucose (g)	0.1[a],1.9[b] (g)	2 g/L
Amino Acids		
L-Arginine	100[b]	100 mg/L
L-Proline	200[b]	200 mg/L
Vitamins		
Thiamine-HCl	2[b]	2 mg/L
Biotin	2[b]	2 mg/L
Nicotinic acid	2[b]	2 mg/L
Calcium pantothenate	2[b]	2 mg/L

Values are expressed in milligrams (per 1 liter of medium) unless noted otherwise. [a] Added before autoclaving. [b] Added after autoclaving.

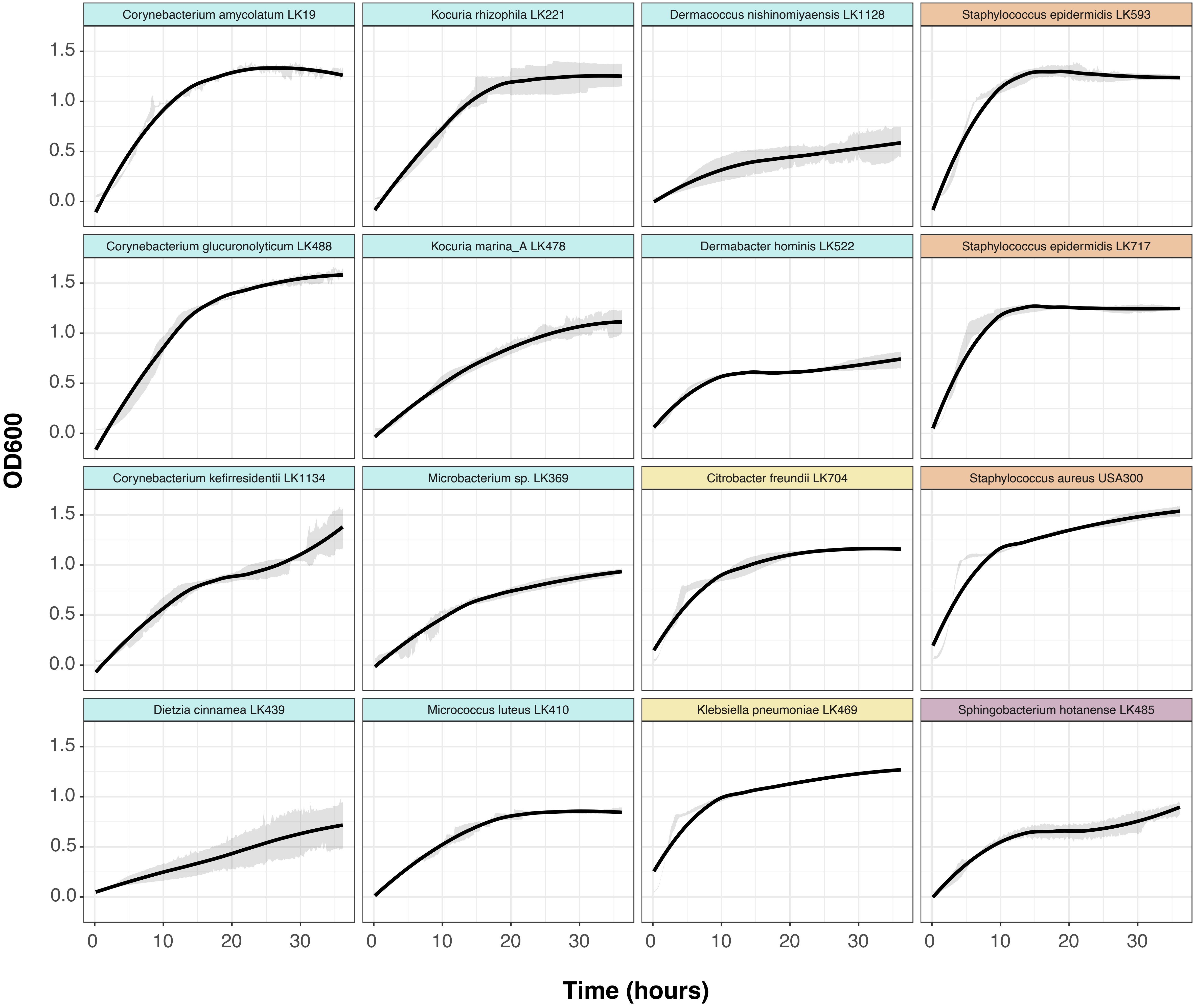

Supplemental Figure 1. For each strain, growth curves were collected in Brain Heart Infusion broth supplemented with 0.2% Tween 80 over 36 hours. Strains are ordered by phylum-level taxonomic assignment. Curves were generated using loess smoothing for averaged OD600 values from at least 2 biological replicates with 3 technical replicates each. Ribbons represent the standard deviation across biological replicates.

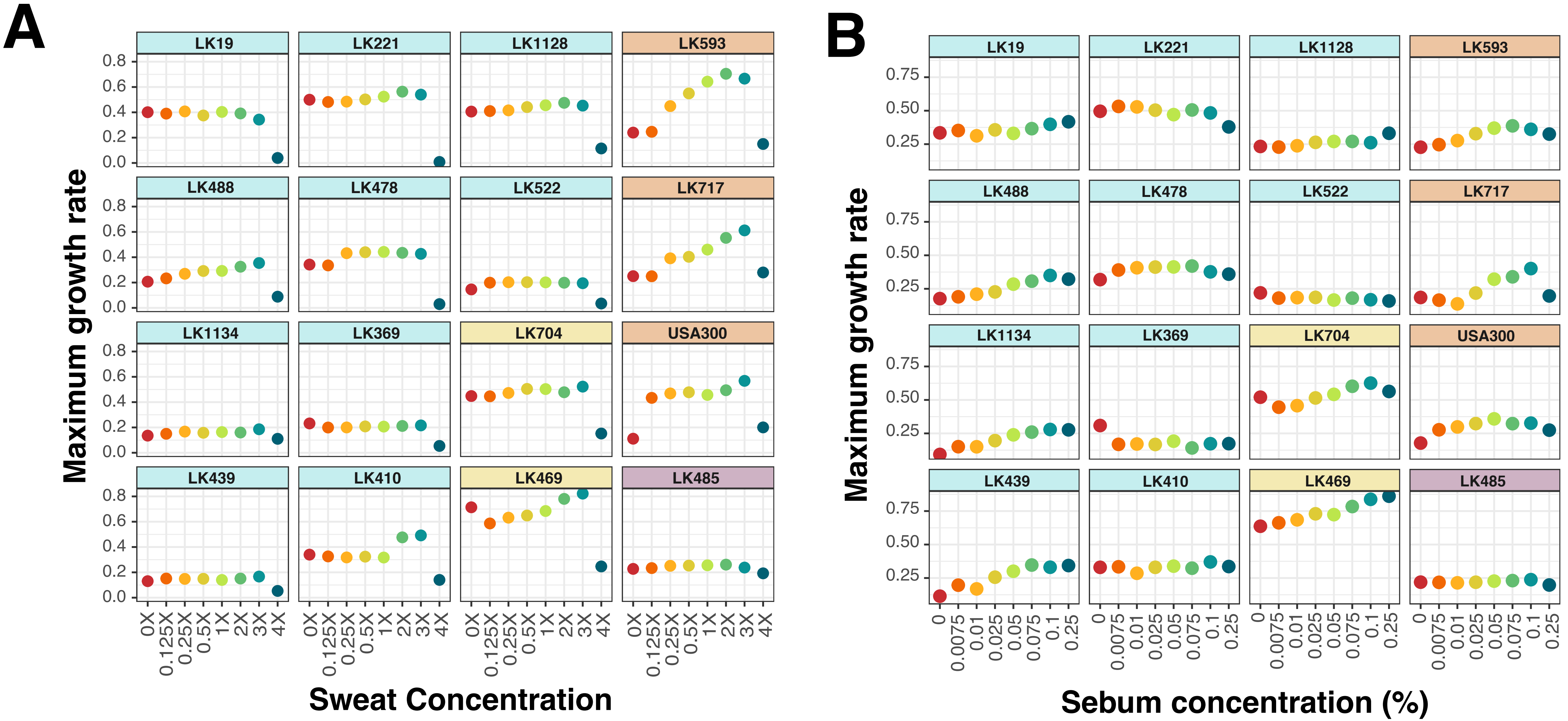

Supplemental Figure 2. A) From the artificial sweat growth curve data, maximum growth rate was calculated for each strain and sweat concentration. B) From the artificial sebum growth curve data, maximum growth rate was calculated for each strain and sebum concentration. Strains are ordered as in Figures 2 and 3.

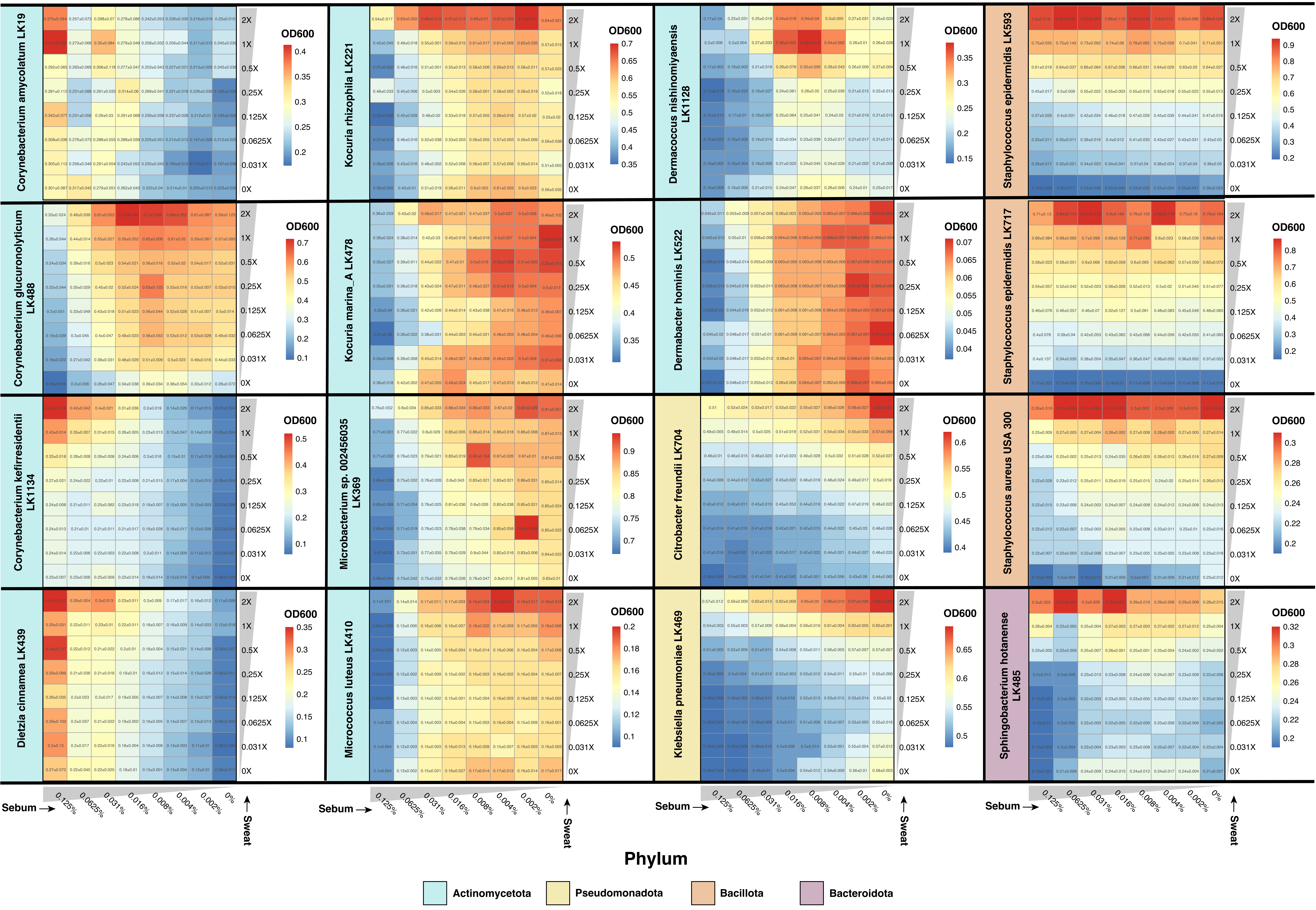

Supplemental Figure 3. Strains were cultured in unique combinations of artificial sweat media (0, 0.031, 0.0625, 0.125, 0.25, 0.5, 1, 2X) and artificial sebum media (0, 0.002, 0.004, 0.008, 0.016, 0.031, 0.0625, 0.125%) for 18 hours, after which an endpoint OD600 reading was recorded. At least three replicates were collected for each strain, and OD600 values from uninoculated media were subtracted from experimental OD600 values. Average OD600 values of the replicates and standard deviation are indicated.

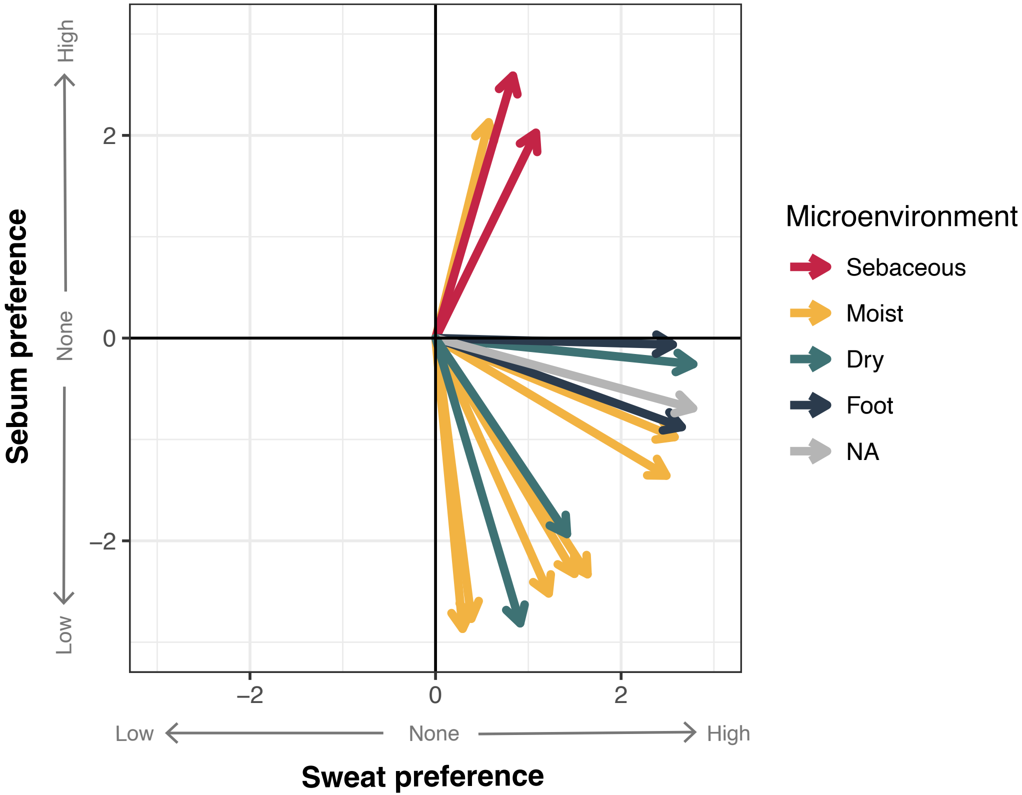

Supplemental Figure 4. Multiple linear regression analysis was performed with the z-score normalized data from the sweat and sebum checkerboard assays. The slope coefficients for sweat concentration and sebum concentration are plotted as an (x,y) coordinate, respectively. Strains are colored by skin microenvironment.
